# Automated computed tomography quantification of fibrosis predicts prognosis in combined pulmonary fibrosis and emphysema in a real-world setting: a single-centre, retrospective study

**DOI:** 10.1186/s12931-020-01545-3

**Published:** 2020-10-20

**Authors:** Masahiro Nemoto, Yuichiro Nei, Brian Bartholmai, Kazuki Yoshida, Hiroki Matsui, Tamao Nakashita, Shinji Motojima, Masahiro Aoshima, Jay H. Ryu

**Affiliations:** 1 Department of Pulmonary Medicine, Kameda Medical Center, Kamogawa, Japan; 2 Department of Immunology, Graduate School of Medicine, Chiba University, 1-8-1 Inohana, Chuo Ward, Chiba, Japan; 3 Department of Rheumatology, Teikyo University Chiba Medical Center, Ichihara, Japan; 4 Department of Radiology, Mayo Clinic, Rochester, MN USA; 5 Departments of Epidemiology and Biostatistics, Harvard T.H. Chan School of Public Health, Boston, MA USA; 6 Clinical Research Support Division, Kameda Institute for Health Science, Kameda College of Health Sciences, Kamogawa, Japan; 7 Department of Rheumatology, Kameda Medical Center, Kamogawa, Japan; 8 Division of Pulmonary and Critical Care Medicine, Mayo Clinic, Rochester, MN USA

**Keywords:** Pulmonary emphysema, Lung, Fibrosis, Tomography, Cohort studies

## Abstract

**Background:**

Combined pulmonary fibrosis and emphysema (CPFE) is a heterogeneous clinico-radiological syndrome without a consensus definition. There are limited data on the relation between the amount of parenchymal fibrosis and prognosis. In this study, we assessed the prognostic implications of the extent of fibrosis assessed by an automated quantitative computed tomography (CT) technique and the radiological and functional change over time in patients with a broad spectrum of fibrotic interstitial lung diseases (ILDs) encountered in a real-world setting.

**Methods:**

We conducted a single-centre, retrospective study of 228 consecutive patients with CPFE, encountered from 2007 to 2015 at Kameda Medical Center, Chiba, Japan. We investigated the prognostic value of automated CT fibrosis quantification and the subsequent course of CPFE.

**Results:**

Among 228 patients with CPFE, 89 had fibrosis affecting < 5% of their lungs, 54 had 5 to < 10% fibrosis, and 85 had ≥ 10% fibrosis at the time of diagnosis. Lower volume of fibrosis correlated with lower rates of mortality and acute exacerbation (*p* < 0.001). In particular, among those with < 5% fibrosis, only 4.5% died and none experienced acute exacerbation during follow-up, whereas 57.6% and 29.4% of those with ≥ 10% fibrosis experienced death and acute exacerbation, respectively. Although, the ≥ 10% fibrosis group had the poorest overall survival as well as the highest incidence of acute exacerbation, the incidence of decline in pulmonary function tests, change per year in total lung volume, and progression of fibrosis on chest CT was highest in the 5 to < 10% fibrosis group. The Cox proportional hazard model for CPFE progression (defined by composite criteria of death, acute exacerbation, and decline in forced vital capacity or diffusing capacity) showed fibrosis proportion was a risk factor independent of age, sex, smoking pack-years, the Charlson Comorbidity Index, lung cancer, connective tissue disease, and idiopathic pulmonary fibrosis.

**Conclusions:**

Less severe (< 5%) fibrosis at baseline was associated with disease stability and better prognosis compared to more severe fibrosis in CPFE occurring with fibrotic ILDs. Further studies including a validation cohort will be needed.

*Trial Registration* Retrospectively registered.

## Background

Combined pulmonary fibrosis and emphysema (CPFE) is a heterogeneous clinico-radiological syndrome, comprising emphysema in the upper lung and fibrosis in the lower lung. CPFE is strongly associated with cigarette smoking thus, considered a smoking-related interstitial lung disease (ILD) [1, 2]. Natural disease history differs between patients with CPFE and those with either pulmonary fibrosis or emphysema alone. The median survival time reported for patients with CPFE varies from 2.1 to 8.5 years, with an increased incidence of pulmonary hypertension and lung cancer [1]. A recent analysis revealed that patients with CPFE had a similar mortality rate with those with idiopathic pulmonary fibrosis (IPF) alone; however, it excluded patients with other fibrotic ILDs [3]. Although initial reports of CPFE cases were described in patients with IPF [4], recent studies included fibrotic ILDs with computed tomography (CT) findings inconsistent with a usual interstitial pneumonia (UIP) pattern [5]. In the absence of a consensus definition for CPFE, it seems important to analyse all patients with co-existent emphysema and fibrotic ILDs (rather than just IPF/UIP) seen on high resolution CT to clarify the prognostic implications of CPFE in the broader population [2].

Study populations in previous reports varied, and no studies have correlated the long-term evolution of CPFE to baseline CT findings and pulmonary function measures; therefore, the prognostic implications of these studies remain uncertain. This study aimed to assess the prognostic implications of the extent of fibrosis assessed by an automated quantitative CT technique and pulmonary function measures at the time of initial CPFE diagnosis.

## Methods

### Study design and participants

This was a single-centre cohort study designed to assess the prognostic value of fibrosis quantification in patients with CPFE adhered to the Strengthening the Reporting of Observational Studies in Epidemiology Statement [6]. The institutional review board of Kameda Medical Center approved the study and allowed use of the opt-out method, instead of requiring informed consent.

Study participants were retrospectively selected from electronic medical records using a computer-assisted search function of Kameda Medical Center, Chiba, Japan, from January 2007 to December 2015. Consecutive adult patients with bilateral fibrosis based on their chest CT radiology report were initially included in the study, instead of using the clinical diagnosis of CPFE, to avoid selection bias. From the chest CT reports, we considered fibrosis cases reported with relevant description, such as: fibrotic changes, ILD, and interstitial pneumonia in Japanese and English. We used the first chest CT scan and pulmonary function tests (PFTs) in the patient-selection period for initial evaluation and the date of diagnosis. In the absence of consensus criteria for CPFE, we avoided using the minimum fibrosis or emphysema criteria and analysed the entire patient cohort with any amount of bilateral lung fibrosis and emphysema to avoid selection bias. The exclusion criteria were: (1) no mention of emphysema in the chest CT report; (2) diagnosis of other specific types of ILD, following the prior publication by Cottin V, et al., such as drug-induced ILD, pneumoconiosis, hypersensitivity pneumonitis, sarcoidosis, pulmonary Langerhans cell histiocytosis, lymphangioleiomyomatosis, or eosinophilic pneumonias [5, 7]; (3) no history of cigarette smoking (to avoid other forms of cystic lung diseases) [2]; and (4) no follow-up chest CT or PFTs > 1 year after the initial evaluation, unless the patient had experienced acute exacerbation or had died.

The lung parenchyma of each eligible patient was automatically segmented from the CT dataset, and each pixel in the lung tissue was classified by the Computer-Aided Lung Informatics for Pathology Evaluation and Rating (CALIPER) [8, 9] software as normal parenchyma; hyperlucent area (moderate or severe low attenuation areas [LAAs]); or fibrosis (ground glass opacity [GGO], reticular densities, or honeycombing pattern). The whole-lung CALIPER results for each participant were classified into three groups based on previous studies [10, 11] as the proportion of fibrosis volume in CPFE and interstitial lung abnormality: (1) < 5%, (2) 5 to < 10%, and (3) > 10% to assess the relationship between the amount of fibrosis and disease progression. We examined the patients’ medical records from January 2007 to December 2018.

### Data collection and CALIPER analysis

The following demographic, clinical, and laboratory data were collected at the time of the initial chest CT study: age, sex, data required for the Charlson Comorbidity Index (CCI) [12] and ILD-gender-age-physiology score [13], smoking history, clinical ILD diagnosis, anti-fibrotic agent use, pulmonary hypertension or lung cancer history, and PFT results. The initial and most recent PFT and chest CT results were collected.

Available chest CT results were evaluated by CALIPER (developed at Mayo Clinic, Minnesota, United States), a system based on histogram signature mapping, and was programmed using expert radiologist analysis of pathologically confirmed datasets, obtained through the Lung Tissue Research Consortium [9, 14], to quantify features of the lung parenchyma. CALIPER classified each lung parenchymal voxel into one of the following categories: normal lung, mild volume of LAAs, moderate LAA, severe LAA, reticular opacities, honeycombing, and GGO. CALIPER algorithm is specifically designed to detect low density pixels that could be within either emphysema or honeycombing [8, 14, 15]. A volumetric morphological analysis of the surrounding area is performed, to determine whether that pixel and its contiguously surrounding pixels should be classified as emphysema or honeycombing. When the algorithm identifies a rounded periphery of higher density surrounding an area of low density, it is classified as a honeycomb cyst; if not, it is classified as emphysema. Fibrosis extent was defined as the sum volume of honeycombing, ground glass, and reticular opacities [9, 15]. The amount of normal lung was defined as the sum volume of normal lung and mild LAAs [16]. Emphysema was defined as the sum volume of moderate and severe LAAs.

### Outcome of interest

The primary outcome was CPFE progression defined by the following criteria: (1) death by any cause, (2) acute exacerbation, (3) decline in forced vital capacity (FVC) > 10% [17], and (4) decline in diffusing capacity of the lung for carbon monoxide (DLco) > 15% [17, 18]. We considered the disease had progressed when any of these criteria were met. The secondary outcome was overall survival calculated from the date of initial chest CT until death; progression-free survival was calculated from the date of the initial chest CT until a patient met any of the progression criteria. Per-year change in PFT and CALIPER quantification based on chest CT was calculated from the interval in the data, divided by the years between the baseline and the most recent test results.

### Statistical analysis

Categorical variables are expressed as number and percentage and were compared using the chi-square and Fisher’s exact tests. Continuous variables are expressed as the median and interquartile range and were compared using the Mann–Whitney *U*-test. We adjusted for potential confounding covariates for the correlation between fibrosis extent and outcome, including age, sex, smoking amount (pack-years), and CCI before data collection to minimise selection bias [12]. Background lung-specific covariates that were adjusted for included lung cancer, connective tissue disease, and IPF. A Cox proportional hazard model for CPFE progression was adjusted by the following additional factors to determine fibrosis proportion: age, sex, pack-years, and CCI. Lung-specific adjustments were for lung cancer, connective tissue disease, and IPF. Overall survival and progression-free survival stratified by fibrosis extent on chest CT were analysed by Kaplan–Meier plots and log-rank tests. Statistical significance was indicated by *p* < 0.05. All analyses were performed using R ver. 3.5.2 (R Foundation for Statistical Computing, Vienna, Austria) [19] with the add-on package EZR ver. 1.36 (Saitama Medical Center, Jichi Medical University, Saitama, Japan) [20].

## Results

### Study participants

**Fig. 1:**
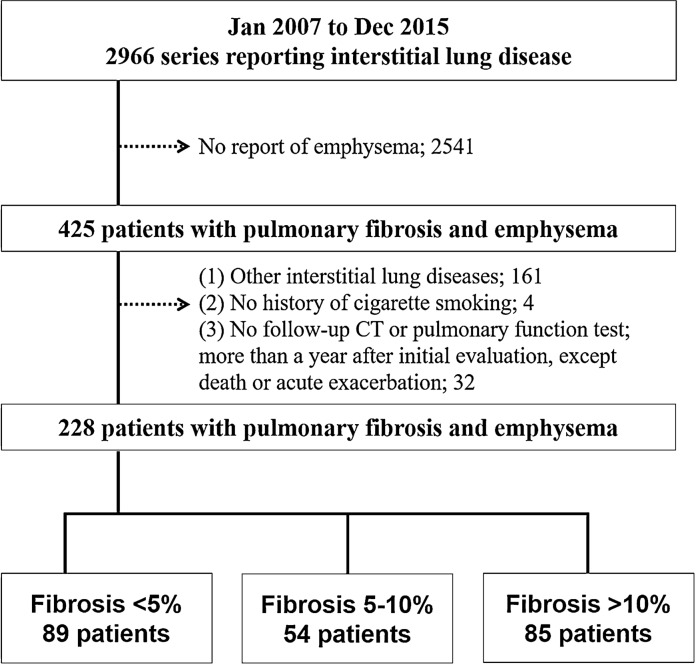
Patient selection flow chart

Figure 1 shows the patient’s selection process. The baseline characteristics of the final 228 patients included in the study are shown in Table 1, and more details are provided in the Additional file 1.

**Table 1:** Baseline patient characteristics

Characteristics	Overall *N* = 228	Fibrosis^a^ < 5%, *N* = 89	Fibrosis 5–10% *N* = 54	Fibrosis ≥ 10% *N* = 85	*p* value^b^
Number of males (%)	205 (89.9)	74 (83.1)	46 (85.2)	85 (100.0)	< 0.001
Median age, years (IQR)	69.9 (66.0, 76.3)	68.0 (62.0, 73.0)	72.0 (67.3, 77.0)	73.0 (66.0, 77.0)	0.001
Median body mass index	22.9 (20.9, 25.0)	22.8 (20.8, 24.8)	23.5 (20.9, 25.9)	22.9 (21.0, 24.6)	0.578
Former/current smoker (%)	155 (68.0)/73(32.0)	52 (58.4)/37(41.5)	39 (72.2)/15(27.8)	64 (75.3)/21(24.7)	0.113
Pack-years smoking, median (IQR)	50 (36, 79)	50 (36, 70)	47 (34, 74)	59 (40, 84)	0.188
Charlson Comorbidity Index score 1/2/3/4/5 ≤ , %	23.2/19.7/25.9/9.6 /21.6	27.0/27.0/22.5/15.7 /7.9	22.2/14.8/25.9/5.6 /31.5	20.0/15.3/29.4/5.9 /29.4	0.002
Serum KL-6, median U/mL (IQR)	554.0 (368.8, 847.5)	367.0 (274.0, 542.0)	626.0 (509.0, 1020.0)	666.0 (452.0, 906.5)	< 0.001
IPF (%)	34 (14.9)	1 (1.1)	10 (18.5)	23 (27.1)	< 0.001
Pulmonary hypertension (%)	26 (11.4)	10 (11.2)	5 (9.3)	11 (12.9)	0.8
Lung cancer (%)	53 (23.2)	11 (12.4)	13 (24.1)	29 (34.1)	0.003
Connective tissue disease (%)	47 (20.6)	24 (27.0)	8 (14.8)	15 (17.6)	0.152
Pulmonary Function Test, median (IQR)					
FEV_1_, L	2.2 (1.8, 2.6)	2.2 (1.8, 2.7)	2.2 (1.8, 2.6)	2.2 (1.8, 2.6)	0.934
FEV_1_, % predicted	96.9 (84.3, 110.3)	94.6 (84.4, 109.1)	102.4 (90.9, 115.2)	96.8 (83.3, 109.4)	0.157
FVC, L	3.13 (2.6, 3.7)	3.32 (2.82, 3.89)	3.15 (2.69, 3.67)	2.94 (2.35, 3.34)	< 0.001
FVC, % predicted	101.2 (86.8, 115.2)	106.1 (96.5, 120.3)	104.1 (91.6, 115.9)	89.4 (76.8, 104.2)	< 0.001
FEV_1_/FVC ratio	70.6 (63.3, 78.7)	66.2 (58.7, 73.2)	70.0 (62.1, 75.0)	77.3 (69.4, 85.4)	< 0.001
DLco, ml/min/mmHg	10.9 (8.0, 13.6)	12.5 (10.3, 16.9)	10.6 (8.9, 13.4)	8.3 (6.2, 12.0)	0.002
DLco, % predicted	66.7 (50.9, 83.3)	82.3 (67.7, 91.3)	71.4 (59.5, 79.7)	53.1 (44.0, 66.9)	< 0.001
Composite physiologic index^c^	27.7 (13.7, 41.1)	11.3 (5.3, 26.0)	27.5 (16.2, 33.6)	42.2 (25.1, 54.3)	< 0.001
ILD-GAP model 0–1/2–3/4–5/6–8, %	11.8/68.0/15.8/4.4	21.3/69.7/9.0/0	5.6/83.3/5.6/5.6	5.9/56.5/29.4/8.2	< 0.001
Initial CT findings^d^					
Total lung volume, median cm^3^ (IQR)	4417.8 (3849.0, 5184.6)	4671.7 (4155.2, 5341.2)	4241.7 (3790.6, 5278.9)	3991.9 (3497.9, 4642.3)	< 0.001
Extent of normal lung, median cm^3^(IQR), %^e^	3104.4 (2359.0, 3781.4), 77.1	3634.1 (3191.6, 4156.0), 82.0	3067.3 (2669.1, 3450.9), 79.9	2467.0 (1853.7, 3049.6), 70.2	< 0.001
Extent of emphysema, median cm^3^ (IQR), %	644.3 (301.0, 1292.1), 15.2	700.6 (330.9, 1424.1), 15.6	630.7 (313.7, 1005.5), 13.1	567.4 (270.8, 1308.6), 13.7	0.394
Extent of fibrosis, median cm^3^ (IQR), %	292.2 (142.0, 528.7), 7.7	133.8 (95.3, 159.5), 2.4	308.9 (275.4, 366.5), 7.0	609.3 (516.4, 780.2), 16.1	< 0.001

*IQR* interquartile range, *IPF* idiopathic pulmonary fibrosis, *FEV 1* forced expiratory volume in one second, *FVC* forced vital capacity, *DLco* diffusing capacity for carbon monoxide

^a^Extent of fibrosis was defined as the percentage (%) sum of reticular shadowing, grand glass opacity, and honeycombing calculated in CALIPER

^b^*p* values are reported for the differences between the fibrosis proportion groups, using a chi-squared test, Fisher exact test, *t* test, or Wilcoxon rank-sum test as appropriate

^c^Composite physiologic index = 91.0 – [0.53 × percent predicted FVC] – [0.65 × percent predicted DLco] + [0.34 × percent predicted FEV1]

^d^Calculated results from CALIPER

^e^Calculated the percentage to account for the emphysema volume and total lung volume in each individual

**Fig. 2:**
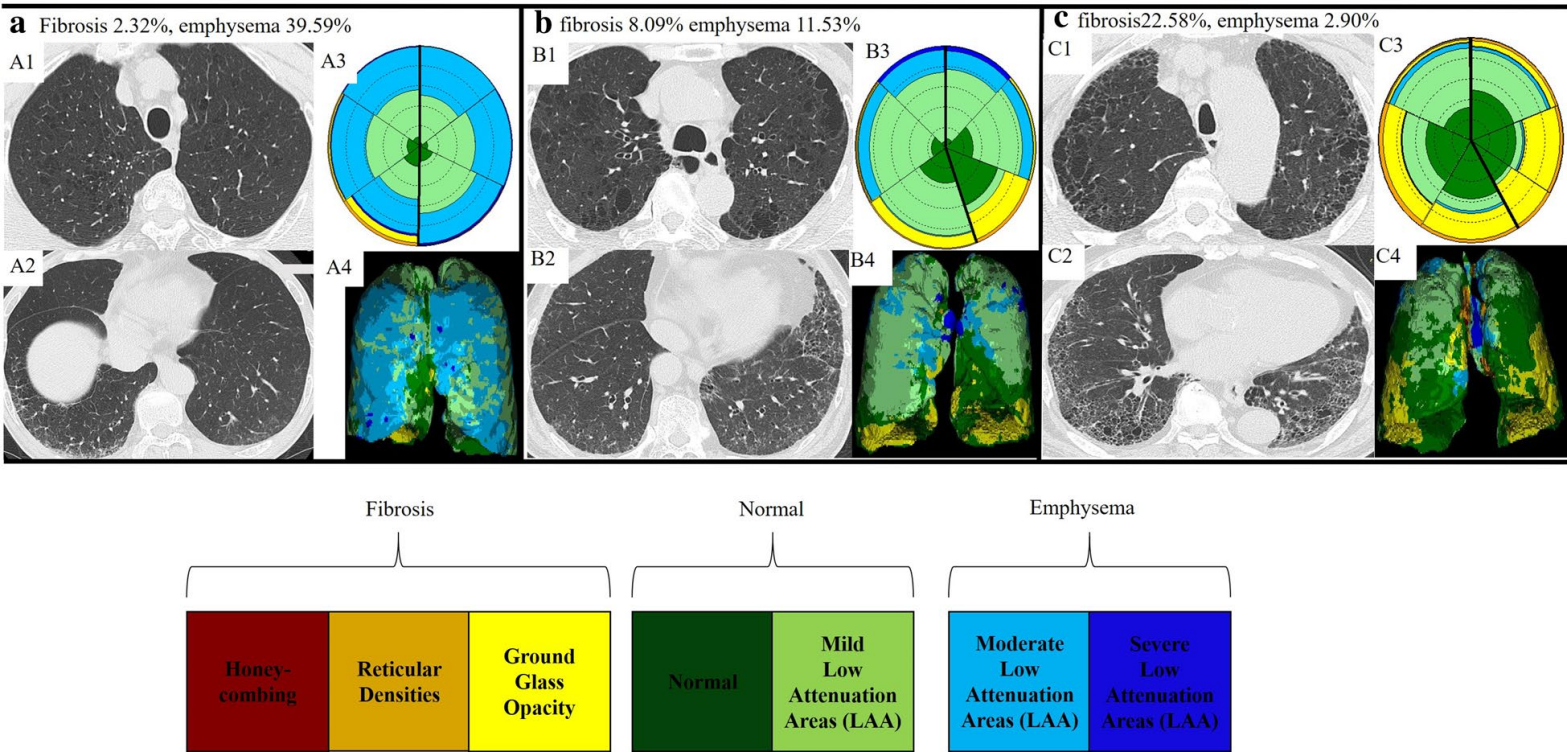
Representative high-resolution computed tomography images from three patients. Images of the upper lung (**a1**, **b1**, and **c1**) and lower lung (**a2**, **b2**, and **c2**) and 3D renderings analysed by computer-aided lung informatics for lung informatics pathology evaluation and rating shown on each right side (**a3**, **4**; **b3**, **4**; and **c3**, **4**)

**Fig. 3:**
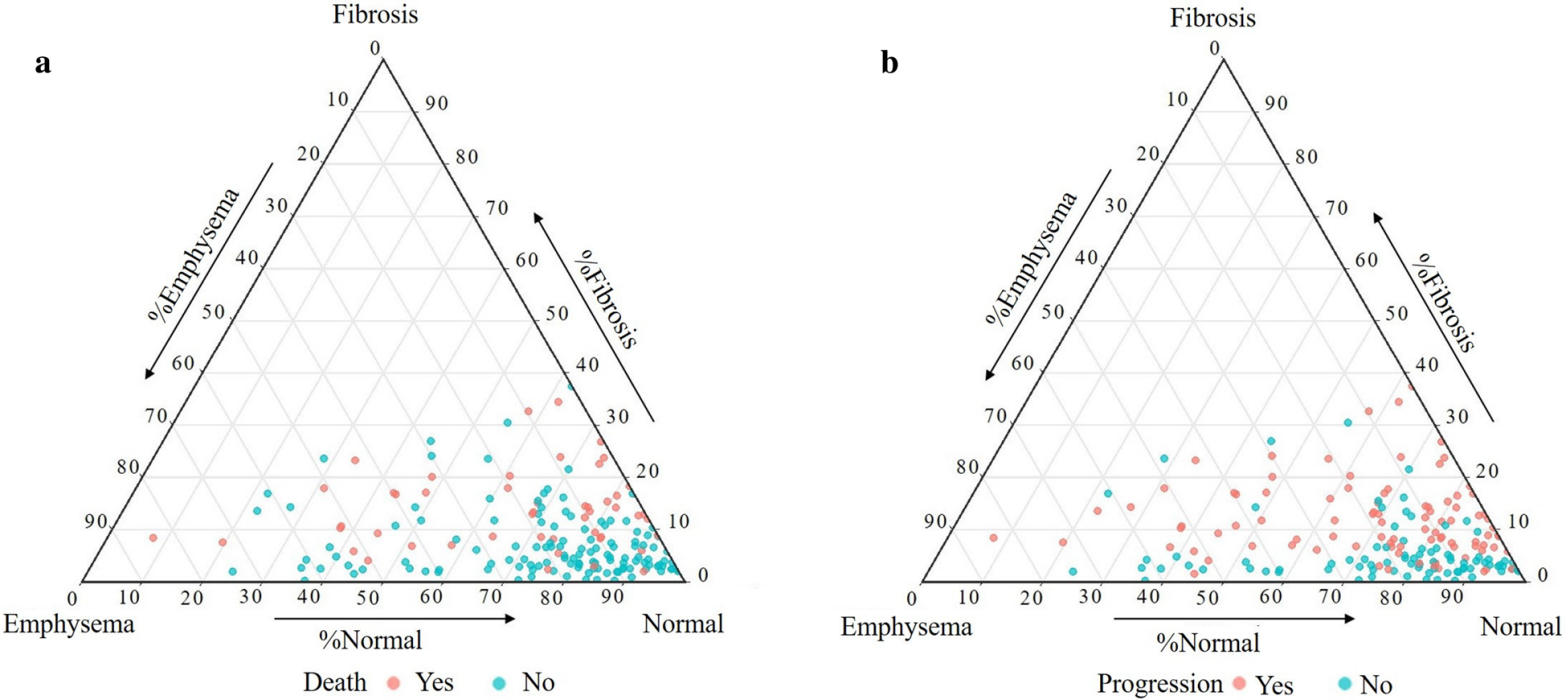
Ternary plot for the extent of fibrosis, emphysema, and normal lung; **a** death vs non-death, **b** progression vs non-progression

Representative images of the CALIPER evaluation are shown in Fig. 2. The ternary plot in Fig. 3 shows the distribution proportion of fibrosis, emphysema, and normal lung for all 228 participants at initial evaluation. Fibrosis was in the range of 30%; however, emphysema was distributed from approximately 0 to 80%. The distribution of the ternary plot from the lower left to the upper right suggests that the percentage of emphysema is negatively correlated to the percentage of fibrosis. Disease progression and death were associated with a higher percentage of fibrosis.

### Outcomes

The study outcomes are shown in Table 2. The numbers of all deaths and acute exacerbations were significantly lower in patients with less severe fibrosis. The composite progression was highest in patients with 5 to < 10% fibrosis compared to other groups (*p* < 0.001). Regarding per-year change in PFTs and chest CT findings, the mean absolute and relative decline in FVC, decline in total lung volume, and progression of fibrosis were highest and lowest in patients with 5 to < 10% fibrosis and < 5% fibrosis, respectively (*p* < 0.001). Per-year change in DLco and emphysema severity was not significantly different across the three groups. The study outcomes in subgroups of emphysema extent are shown in Additional file 2 and none of them were significantly different.

**Table 2:** Study outcomes

	Overall, *N* = 228	Fibrosis^a^ < 5%, *N* = 89	Fibrosis 5–10%, *N* = 54	Fibrosis ≥ 10%, *N* = 85	*p* value ^b^
Composite progression (%)	127 (55.7)	16 (18.0)	42 (77.8)	69 (81.2)	< 0.001
All deaths (%)	75 (32.9%)	4 (4.5)	22 (40.7)	49 (57.6)	< 0.001
Respiratory-related deaths (%)	47 (20.6)	2 (2.2)	14 (25.9)	31 (36.5)	< 0.001
Acute exacerbation (%)	35 (15.4)	0 (0.0)	10 (18.5)	25 (29.4)	< 0.001
Progression in pulmonary function tests (%)	67 (29.4%)	12 (13.5)	31 (57.4)	24 (28.2)	< 0.001
Change in median body mass index/year (IQR)	− 0.02 (− 0.34, 0.26)	0.00 (− 0.15, 0.17)	− 0.10 (− 0.28, 0.17)	− 0.04 (− 0.30, 0.12)	0.103
Smoking cessation during the follow-up period (%)	40 (17.5)	18 (50.0)	9 (60.0)	13 (59.1)	0.718
Changes in forced vital capacity^c^					
Median absolute change/year, L (IQR)	− 0.06 (− 0.15, 0.02)	− 0.02 (− 0.07, 0.04)	− 0.12 (− 0.23, − 0.08)	− 0.07 (− 0.16, 0.06)	< 0.001
Median relative change/year, % (IQR)	− 1.67 (− 4.57, 0.66)	− 2.88 (− 8.41, 3.08)	− 13.66 (− 22.01, − 5.56)	− 4.30 (− 15.27, 7.87)	< 0.001
Changes in DLco					
Median absolute change/year, ml/min/mmHg (IQR)	− 0.25 (− 0.49, 0.11)	− 0.05 (− 0.24, 0.64)	− 0.93 (− 2.46, − 0.08)	− 0.31 (− 0.46, 0.00)	0.057
Median relative change/year, % (IQR)	3.03 (1.72, 5.60)	1.46 (− 0.91, 3.90)	− 3.90 (− 21.74, − 0.09)	− 1.36 (− 2.02, 1.36)	0.05
Changes in composite physiologic index^d^					
median absolute change/year (IQR)	0.93 (− 5.54, 10.11)	0.55 (− 5.08, 5.43)	1.33 (− 5.73, 12.48)	0.28 (− 4.98, 7.58)	0.86
Changes in computed tomography findings, median, %/year (IQR)					
Total lung volume	− 6.97 (− 16.91, − 0.15)	− 3.69 (− 10.11, 1.32)	− 12.87 (− 24.13, − 2.95)	− 11.46 (− 21.46, − 2.64)	< 0.001
Extent of normal lung	− 1.69 (− 4.08, 0.53)	− 1.21 (− 2.57, − 0.47)	− 2.21 (− 4.09, − 1.35)	− 2.11 (− 5.48, − 0.50)	0.083
Extent of emphysema	0.68 (− 0.01, 1.92)	0.49 (− 0.01, 1.77)	0.70 (0.01, 1.50)	0.96 (− 0.02, 2.43)	0.505
Extent of fibrosis	1.57 (0.10, 3.85)	0.21 (− 0.03, 0.91)	1.64 (0.33, 4.05)	0.89 (0.11, 4.14)	0.001

*IQR* interquartile range, *FVC* forced vital capacity, *DLco* diffusing capacity for carbon monoxide

^a^Extent of fibrosis was defined the extent (%) of combined reticular shadow, grand glass opacity, and honeycombing resulted in CALIPER. Twenty-nine CT series were unavailable for CALIPER analysis because of CT condition

^b^*P* values are reported for the difference between groups between fibrosis proportions, using a × 2 test, Fisher exact test, *t* test, or Wilcoxon rank-sum test as appropriate

^c^Change in FVC was calculated as the relative change (FVC baseline − FVC timepoint 2/FVC baseline, using either FVC in litres or % predicted FVC) and absolute change (FVC baseline − FVC timepoint 2, using % predicted FVC)

^d^Composite physiologic index = 91.0 − [0.53 × percent predicted FVC] − [0.65 × percent predicted DLco] + [0.34 × percent predicted FEV1]

**Fig. 4:**
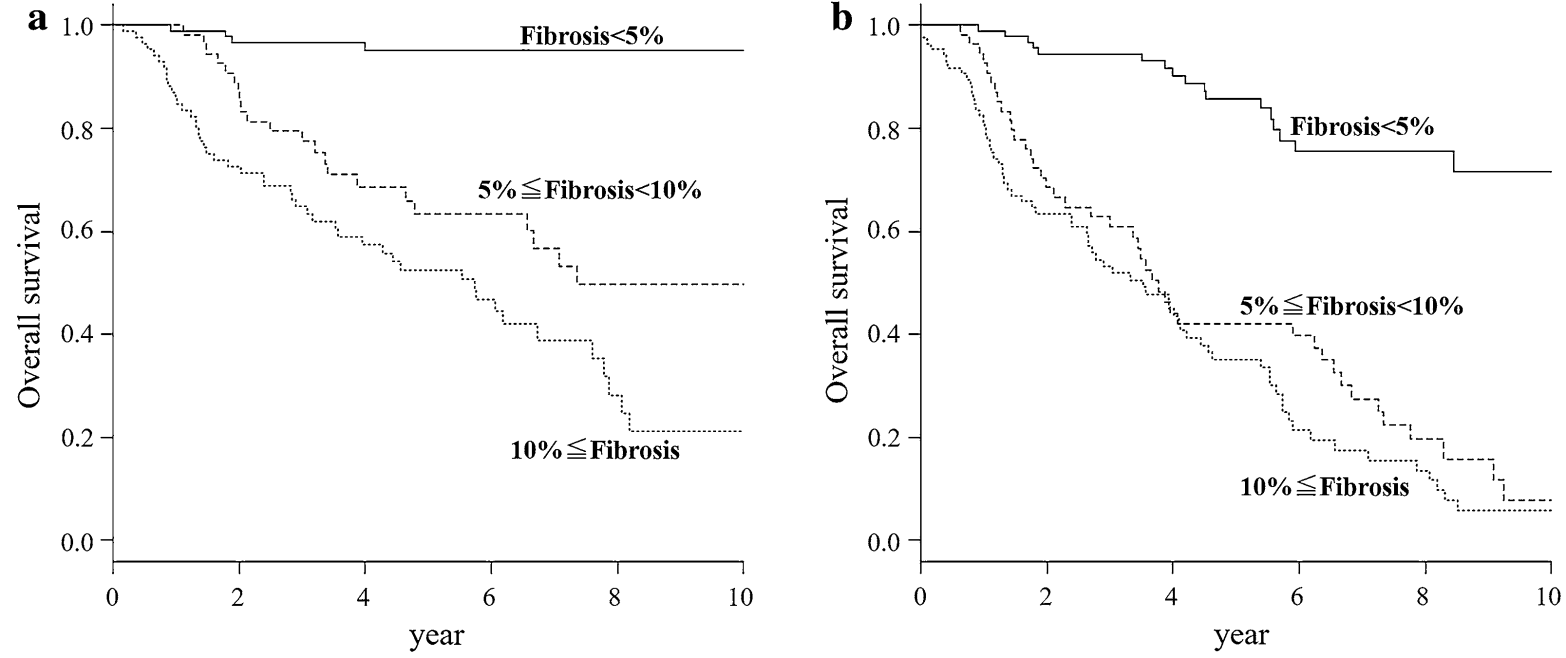
Kaplan–Meier curve for **a** overall survival and **b** progression-free survival. Line: patients with < 5% fibrosis. Dash: patients with 5 to < 10% fibrosis. Dot: patients with ≥ 10% fibrosis

Kaplan–Meier curves for overall survival and progression-free survival are shown in Fig. 4. Overall survival and progression-free survival significantly differed across the fibrosis groups. Overall survival and progression-free survival were 7.3 years (interquartile range [IQR]: 4.8 years–not applicable) and 3.7 years (IQR: 2.7–6.4 years), respectively, in the 5 to < 10% fibrosis group; and 5.7 years (IQR: 3.5–7.6 years) and 3.5 years (IQR: 2.4–4.2 years), respectively, in the ≥ 10% group. For the < 5% fibrosis group, overall survival could not be statistically calculated because the events were below 50%. In contrast, Kaplan–Meier curves for PFT progression showed the 5 to < 10% fibrosis group and the < 5% fibrosis group were most and least affected, respectively. Cox proportional hazard model for CPFE progression was used to assess fibrosis proportion (Table 3). In hazard models, fibrosis proportion was a risk factor independent of age, sex, smoking pack-years, CCI, lung cancer, connective tissue disease, and IPF.

**Table 3:** Cox regression model for progression (vs. fibrosis < 5%)

	Fibrosis 5–10%		Fibrosis ≥ 10%	
	HR (95% CI)	*p* value	HR (95% CI)	*p* value
Fibrosis proportion only	7.42 (3.31, 16.65)	< 0.001	5.61 (2.47, 12.74)	< 0.001
Adjusted for age and sex	7.42 (3.28, 16.79)	< 0.001	5.76 (2.49, 13.35)	< 0.001
Adjusted for age, sex, pack-years smoking, and CCI	7.58 (3.34, 17.24)	< 0.001	6.24 (2.65, 14.71)	< 0.001
Adjusted for age, sex, pack-years smoking, CCI, lung cancer, and CTD	7.49 (3.27, 17.16)	< 0.001	6.17 (2.60, 14.63)	< 0.001
Adjusted for age, sex, pack-years smoking, CCI, lung cancer, CTD, and IPF	6.85 (2.95, 15.89)	< 0.001	5.78 (2.42, 13.85)	< 0.001

Hazard ratios are shown (with 95% confidence intervals in parentheses)

*CCI* Charlson Comorbidity Index, *CTD* connective tissue disease, *IPF* idiopathic pulmonary fibrosis, *CI* confidence interval

### Comparison of patients with and without connective tissue disease

Since 20.6% of the study population had connective tissue diseases (CTDs), we performed subgroup analysis to compare characteristics and outcomes in patients with CTD versus those without CTD. The CTD group comprised significantly more female patients, of younger age, with fewer smoking pack-years, and lower lung cancer prevalence; CT findings and fibrosis quantity did not differ significantly (Additional file 3). Most patients with CTD (~ 60%) had rheumatoid arthritis. Among those without CTD, nearly 25% manifested positive autoantibody, consistent with the concept of interstitial pneumonia with autoimmune features formulated in 2015 [21]. Kaplan–Meier curves for overall survival showed no significant difference between the subgroups (Additional file 4). Multivariate analysis (Additional file 5) revealed mortality was not influenced by CTD or sex; instead, it was affected by the presence of IPF, lung cancer, and age.

## Discussion

This study aimed to elucidate the relation between the baseline amount of fibrosis quantified by an automated CT technique and the subsequent course of patients with CPFE. Our results show progression in patients with baseline fibrosis < 5% was not as prominent as in the other CPFE groups and the ≥ 10% fibrosis group had the poorest overall survival as well as the highest incidence of acute exacerbation. Baseline amount of fibrosis was an independent and strong predictor of prognosis. In contrast, in terms of progression-free survival, 5 to < 10% fibrosis group showed a similar Kaplan–Meier curve compared to the ≥ 10% fibrosis group. The incidence of composite progression (especially decline in PFTs), change per year in total lung volume, and progression of fibrosis on chest CT was highest in the 5 to < 10% fibrosis group.

Although Choi SH et al. reported fibrosis extent to correlate with prognosis of CPFE, it was not identified to be an independent prognostic factor [22]. In the Cox regression analysis, we considered the following factors: age, sex, and amount of cigarette smoking, which were previously reported to be associated with emphysema, interstitial lung abnormality, and disease progression by a sub-analysis of the Framingham Heart Study [11, 23]. The CCI [12] was also included in the regression analysis. Finally, lung-specific variables were entered into the models such as lung cancer and IPF, both of which have been identified to be independent poor prognostic factors [24], and CTD-ILD, another common cause of CPFE [25, 26]. Our results revealed that age and male sex were associated with an increased amount of fibrosis. Moreover, patients with CPFE had a high prevalence of lung cancer (23.2%), CTD (20.6%), and IPF (14.9%). It appears that our modelling was appropriate when considering the results of patient characteristics in the current study. Given our Cox regression analysis, we concluded that the extent of fibrosis was an independent and strong prognostic factor for patients with CPFE.

Our subgroup analysis comparing patients with CTD and those without showed that the majority of CTD was rheumatoid arthritis and many others with CPFE had autoantibodies related to rheumatoid arthritis, reinforcing the potential role of cigarette smoking in both diseases [27, 28].

Most previous studies have investigated CPFE within the IPF population rather than investigate the broader CPFE population including those with other fibrotic ILDs, or analysed only patients above a certain threshold of fibrosis extent [5, 29]. To the best of our knowledge, our study included the largest cohort of patients with CPFE with the full spectrum of fibrosis extent encountered in the real-world setting, adding evidence regarding the independent prognostic value of fibrosis quantification.

Although Wiggins et al. [4] first reported combined cryptogenic fibrosing alveolitis and emphysema in eight cigarette smokers in the 1980s, with a unique presentation of severe breathlessness and low gas transfer without apparent airflow obstruction; CPFE remained poorly recognised until the 2000s [5]. In 2010, many studies conducted on CPFE in IPF recognised a poor prognosis due to the high prevalence of pulmonary hypertension and lung cancer [2, 5, 30]; the current study also showed that IPF is associated with poor outcome. Alsumrain et al. reported a similar proportion of IPF to that in our study in their CPFE cohort [10], and the remaining patients were either unclassifiable or diagnosed with secondary ILD using clinico-radiological characteristics. This suggests that careful assessment is needed to identify patients who may benefit from lung biopsy to diagnose UIP patterns or another potentially progressive ILD. We might consider such an intensive approach especially for patients with 5 to < 10% fibrosis who apparently showed radiological and functional progression, yet had less mortality and acute exacerbation than the > 10% fibrosis group. Conversely, patients with CPFE with < 5% showed stable disease behaviour for up to 10 years. We might say that the patients with < 5% do not require monitoring as closely as that required for patients with > 5% fibrosis.

This study has several limitations. First, its single-centre, retrospective design may limit external validity. However, an advantage of our general and tertiary hospital was that over 80% of board-certified pulmonary physicians were within a 50 km radius, making it possible to follow-up patients with multiple assessments over long periods, similar to that in a population-based study. Second, the inclusion and exclusion criteria are debatable. We included any proportion of emphysema and fibrosis. According to the American Thoracic Society/European Respiratory Society 2013 statement of idiopathic interstitial pneumonias (IIP) [29], CPFE was classified as a smoking-related IIP. Thus, there is no global consensus on the definition of CPFE. The number of excluded non-smokers, as the result of current analysis, were only 4. Thus, no impact on the results is expected. In addition, the exclusion criteria referred from the past literature [5] made easy to compare between studies, and it also made possible to exclude the other cystic diseases such as Langerhans cell histiocytosis possibly occurring as the other cystic diseases of smokers. Third, the definition of progression is a matter of debate. Cottinet et al. published a pooled analysis reporting that FVC measurements might be inappropriate for monitoring disease progression in patients with IPF and emphysema extent greater than or equal to 15% [31]. Certainly, our results for CPFE showed discrepancy in survival and progression in patients > 10% fibrosis. However, FVC remains the standard for evaluating disease progression. Further study is necessary to improve the prognostic predictability of CPFE. Finally, CT scans were obtained for clinical reasons and not at regular intervals as part of a prospective study; thus, there may have been more intensive follow-up in participants with more rapid disease progression. Automated quantification of CT data also might be made some difficulty on generalizability.

## Conclusion

Patients with < 5% fibrosis showed relatively benign disease behaviour in terms of both overall survival and functional preservation. However, careful follow-up may be required for patients with > 5% fibrosis, and especially when the extent exceeds 10%. Further studies are required to validate the classification of CPFE.

## Supplementary information

**Additional file 1.** Detailed baseline characteristics.

**Additional file 2.** Study outcomes in patients with emphysema subgrouping.

**Additional file 3.** Baseline characteristics in patients with or without connective tissue disease.

**Additional file 4.** Kaplan–Meier curve for overall survival. Line: patients without connective tissue disease. Dash: patients with connective tissue disease.

**Additional file 5.** Predictors of mortality by logistic regression analysis.

## Data Availability

Not applicable.

## Abbreviations

CALIPER: Computer-Aided Lung Informatics for Pathology Evaluation and Rating
CCI: Charlson Comorbidity Index
CPFE: Combined pulmonary fibrosis and emphysema
CT: Computed tomography
CTD: Connective tissue diseases
DLco: Diffusing capacity of the lung for carbon monoxide
FVC: Forced vital capacity
GGO: Ground glass opacity
IIP: Idiopathic interstitial pneumonias
ILD: Interstitial lung disease
IPF: Idiopathic pulmonary fibrosis
LAAs: Low attenuation areas
PFT: Pulmonary function tests
UIP: Usual interstitial pneumonia
