# Supplementary material for: Automated computed tomography quantification of fibrosis predicts prognosis in combined pulmonary fibrosis and emphysema in a real-world setting: a single-centre, retrospective study
Source: Respir Res. 2020 Oct 20;21:275. doi: 10.1186/s12931-020-01545-3 (PMC7576807; doi:10.1186/s12931-020-01545-3)
Supplement: Supplementary file 1 — Additional file 1. Detailed baseline characteristics. [file 12931_2020_1545_MOESM1_ESM.docx]

***Study participants***

Among 2966 consecutive CT reports with bilateral pulmonary fibrosis during the study period, emphysema was not noted in 2541 additional patient reports, leaving 425 patients with CPFE (Figure 1). Subsequently, 161 patients were excluded for other non-fibrotic ILDs, 4 patients did not have history of cigarette smoking, and 32 patients had not undergone chest CT or PFTs for more than a year after initial evaluation, not because of acute exacerbation or death. Finally, 228 patients with CPFE were included in this study. To summarize the CT scan techniques, 98% of scans were acquired with the Aquilion series (Toshiba, Japan), with peak voltage of 120 kV (range, 50–708 mA), 83% with 2.0 mm slice thickness, and 81% with FC13 kernel.

Baseline characteristics of the 228 patients are summarized in Table 1 and were compared according to fibrosis severity. Male sex, older age, serum KL-6 level, and IPF proportion were associated with higher degree of fibrosis. As the amount of fibrosis increased, baseline FVC and DLco significantly decreased and the forced expiratory volume in one second divided by FVC and the composite physiologic index significantly increased. In the initial CT findings, the total lung volume and extent of normal lung were significantly lower with increasing fibrosis. In contrast, the volume of emphysema was not significantly different in any of the groups.
