# Supplementary material for: Automated computed tomography quantification of fibrosis predicts prognosis in combined pulmonary fibrosis and emphysema in a real-world setting: a single-centre, retrospective study
Source: Respir Res. 2020 Oct 20;21:275. doi: 10.1186/s12931-020-01545-3 (PMC7576807; doi:10.1186/s12931-020-01545-3)
Supplement: Supplementary file 2 — Additional file 2. Study outcomes in patients with emphysema subgrouping. [file 12931_2020_1545_MOESM2_ESM.docx]

| \|  \| **Emphysema^*^<10%, N= 80** \| **Emphysema 10-20%, N= 65** \| **Fibrosis ≥20%, N= 83** \| ***p* value** ^†^ \| \| --- \| --- \| --- \| --- \| --- \| \| Composite progression (%) \| 49 (61.3) \| 31 (47.7) \| 47 (56.6) \| <0201 \| \| All deaths (%) \| 32 (40.0) \| 15 (23.2) \| 28 (33.7) \| <0.096 \| \| Respiratory-related deaths (%) \| 19 (23.8) \| 10 (15.4) \| 18 (21.7) \| <0.444 \| \| Acute exacerbation (%) \| 13 (16.2) \| 13 (20.0) \| 9 (10.8) \| <0.297 \| \| Progression in pulmonary function tests (%) \| 25 (31.2) \| 21 (32.3) \| 28 (25.3) \| <0.586 \| |
| --- | --- | --- | --- | --- | --- | --- | --- | --- | --- | --- | --- | --- | --- | --- | --- | --- | --- | --- | --- | --- | --- | --- | --- | --- | --- | --- | --- | --- | --- | --- |

^*^ Extent of emphysema was defined the extent (%) of combined moderate and severe low attenuation areas resulted in CALIPER.

In those subgroups contained similar number of patients (%) with fibrosis ≥10%; 31 (38.8%), 22 (33.8), and 32 (38.6), respectively from the least emphysema. (*p* value = 0.593)

^†^ P values are reported for the difference between groups between fibrosis proportions, using a x 2 test.
