# Supplementary material for: Automated computed tomography quantification of fibrosis predicts prognosis in combined pulmonary fibrosis and emphysema in a real-world setting: a single-centre, retrospective study
Source: Respir Res. 2020 Oct 20;21:275. doi: 10.1186/s12931-020-01545-3 (PMC7576807; doi:10.1186/s12931-020-01545-3)
Supplement: Supplementary file 3 — Additional file 3. Baseline characteristics in patients with or without connective tissue disease. [file 12931_2020_1545_MOESM3_ESM.docx]

| \| **Characteristics** \| **CPFE with CTD**  **N=47** \| **CPFE without CTD**  **N=181** \| **P value**^*^ \| \| --- \| --- \| --- \| --- \| \| Number of male patients (%) \| 32 (68.1) \| 173 (95.6) \| <0.001 \| \| Median age-years (IQR) \| 68.0 (60.0, 70.5) \| 71.0 (66.0, 77.0) \| <0.001 \| \| Former/Current smoker (%) \| 26 (55.3)/21 (44.7) \| 129 (71.3)/52 (28.7) \| 0.080 \| \| Pack-years smoking, median (IQR) \| 45 (28, 68.8) \| 51 (40, 80) \| 0.035 \| \| Serum KL-6, median U/mL (IQR) \| 534 (308, 844) \| 564 (383, 851) \| 0.430 \| \| Pulmonary hypertension (%) \| 7 (14.9) \| 19 (10.5) \| 0.440 \| \| Lung cancer (%) \| 3 (6.4) \| 50 (27.6) \| 0.002 \| \| Connective tissue disease \|  \|  \|  \| \| Rheumatoid arthritis (%) \| 28 (59.6) \|  \|  \| \| Systemic sclerosis (%) \| 6 (12.8) \|  \|  \| \| ANCA-associated vasculitis (%) \| 4 (8.5) \|  \|  \| \| Dermatomyositis (%) \| 4 (8.5) \|  \|  \| \| Systemic Lupus Erythematosus (%) \| 4 (8.5) \|  \|  \| \| Sjogren’s syndrome (%) \| 1 (2.1) \|  \|  \| \| Serological autoimmunity (%)**^†^** \| 36 (76.6) \| 45 (24.5) \|  \| \| Rheumatoid factor (%) \| 25 (53.1) \| 20 (11.0) \|  \| \| Anti-citrullinated protein antibody (%) \| 15 (31.9) \| 15 (8.3) \|  \| \| Anti-nuclear antibody (%) \| 14 (29.8) \| 20 (11.0) \|  \| \| Initial CT findings^‡^ \|  \|  \|  \| \| Total lung volume, median cm^3^ (IQR) \| 4208.1 (3967.5, 5062.7) \| 4418.3 (3787.4, 5250.3) \| 0.919 \| \| Extent of normal lung, median cm^3^ (IQR) %^j^ \| 3035.6 (2804.0, 3724.4), 76.4 \| 3191.6 (2335.4, 3824.9), 76.3 \| 0.953 \| \| Extent of emphysema, median cm^3^ (IQR) % \| 693.1 (251.4, 1361.3), 17.0 \| 574.6 (265.1, 1194.0), 13.8 \| 0.437 \| \| Extent of fibrosis, median cm^3^ (IQR) % \| 173.1 (81.7, 532.4), 4.2 \| 301.2 (143.5, 523.3), 6.8 \| 0.089 \| \| Extent of fibrosis \|  \|  \| 0.161 \| \| Fibrosis <5% (%) \| 24 (51.1) \| 65 (35.9) \|  \| \| Fibrosis 5–10% (%) \| 8 (17.0) \| 46 (25.4) \|  \| \| Fibrosis ≥10% (%) \| 15 (31.9) \| 70 (38.7) \|  \| |
| --- | --- | --- | --- | --- | --- | --- | --- | --- | --- | --- | --- | --- | --- | --- | --- | --- | --- | --- | --- | --- | --- | --- | --- | --- | --- | --- | --- | --- | --- | --- | --- | --- | --- | --- | --- | --- | --- | --- | --- | --- | --- | --- | --- | --- | --- | --- | --- | --- | --- | --- | --- | --- | --- | --- | --- | --- | --- | --- | --- | --- | --- | --- | --- | --- | --- | --- | --- | --- | --- | --- | --- | --- | --- | --- | --- | --- | --- | --- | --- | --- | --- | --- | --- | --- | --- | --- | --- | --- | --- | --- | --- | --- | --- | --- | --- | --- | --- | --- | --- | --- | --- | --- | --- | --- | --- | --- | --- | --- | --- | --- | --- | --- |

*CPFE* combined pulmonary fibrosis and emphysema, *CTD* connective tissue disease, *IQR* interquartile range, *CT* computed tomography.

^*^ P values are reported for the differences between the fibrosis proportion groups, using a chi-squared test, Fisher exact test, t test, or Wilcoxon rank-sum test as appropriate.

**^†^** Each positivity was defined as follows: rheumatoid factor ≥30 U/ml, anti-citrullinated protein antibody≥4.5 EU/mL, ANA≥1:320 titer.

^‡^Calculated results from CALLIPER.
