# Supplementary figures and images for: Automated computed tomography quantification of fibrosis predicts prognosis in combined pulmonary fibrosis and emphysema in a real-world setting: a single-centre, retrospective study
Source: Respir Res. 2020 Oct 20;21:275. doi: 10.1186/s12931-020-01545-3 (PMC7576807; doi:10.1186/s12931-020-01545-3)

**
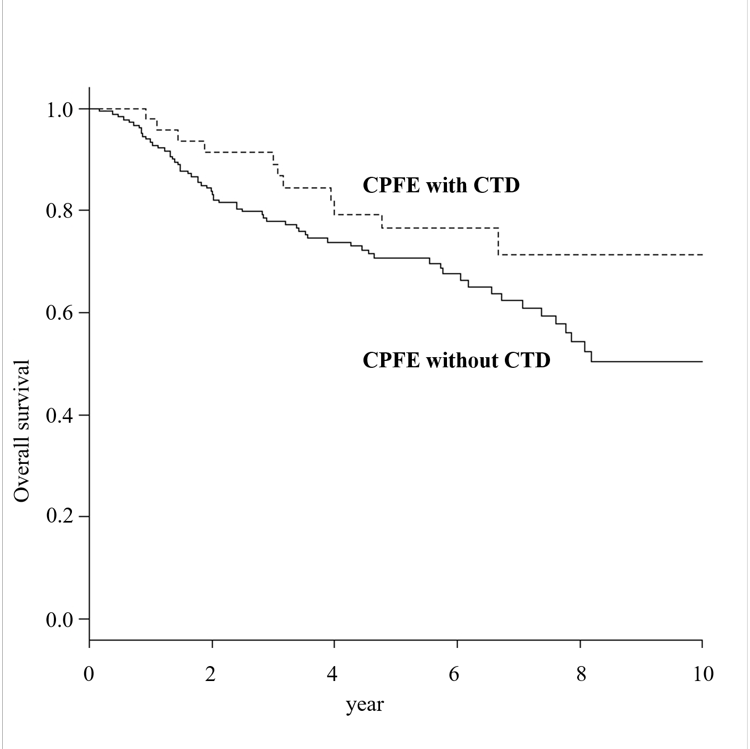
**

Supplement: Supplementary file 4 — Additional file 4. Kaplan–Meier curve for overall survival. Line: patients without connective tissue disease. Dash: patients with connective tissue disease. [file 12931_2020_1545_MOESM4_ESM.docx]
