# Supplementary material for: Automated computed tomography quantification of fibrosis predicts prognosis in combined pulmonary fibrosis and emphysema in a real-world setting: a single-centre, retrospective study
Source: Respir Res. 2020 Oct 20;21:275. doi: 10.1186/s12931-020-01545-3 (PMC7576807; doi:10.1186/s12931-020-01545-3)
Supplement: Supplementary file 5 — Additional file 5. Predictors of mortality by logistic regression analysis. [file 12931_2020_1545_MOESM5_ESM.docx]

| \| **Variables** \| **Odds ratio** \| **95%CI**^*^ \| **P value** \| \| --- \| --- \| --- \| --- \| \| Age \| 1.08 \| 1.04–1.13 \| <0.001 \| \| Sex, male \| 0.91 \| 0.28–2.91 \| 0.87 \| \| Connective tissue disease \| 1.60 \| 0.68–3.81 \| 0.285 \| \| Idiopathic pulmonary fibrosis \| 2.44 \| 1.07–5.58 \| 0.034 \| \| Lung cancer \| 3.25 \| 1.63–6.49 \| <0.001 \| |
| --- | --- | --- | --- | --- | --- | --- | --- | --- | --- | --- | --- | --- | --- | --- | --- | --- | --- | --- | --- | --- | --- | --- | --- | --- |

^*^ 95%*CI* 95% confidence interval.
